# Minimally invasive surgery vs. open thoracotomy for non-small-cell lung cancer with N2 disease: a systematic review and meta-analysis

**DOI:** 10.3389/fmed.2023.1152421

**Published:** 2023-05-31

**Authors:** Songlin Liu, Shaopeng Li, Yong Tang, Rixin Chen, Guibin Qiao

**Affiliations:** ^1^Department of Thoracic Surgery, Guangdong Provincial People's Hospital (Guangdong Academy of Medical Sciences), Southern Medical University, Guangzhou, China; ^2^The Second School of Clinical Medicine, Southern Medical University, Guangzhou, China; ^3^Department of Thoracic Surgery, The Ninth People's Hospital of Shenzhen, Shenzhen, China

**Keywords:** robotic-assisted thoracoscopic surgery, video-assisted thoracoscopic surgery, non-small cell lung cancer, N2 disease, minimally invasive surgery

## Abstract

**Background:**

This meta-analysis aimed to investigate the effectiveness and safety of minimally invasive surgery [MIS, including robotic-assisted thoracoscopic surgery (RATS) and video-assisted thoracoscopic surgery (VATS)] and open thoracotomy (OT) for non-small cell lung cancer (NSCLC) patients with N2 disease.

**Methods:**

We searched online databases and studies from the creation of the database to August 2022, comparing the MIS group to the OT group for NSCLC with N2 disease. Study endpoints included intraoperative outcomes [e.g., conversion, estimated blood loss (EBL), surgery time (ST), total lymph nodes (TLN), and R0 resection], postoperative outcomes [e.g., length of stay (LOS) and complication], and survival outcomes [e.g., 30-day mortality, overall survival (OS), and disease-free survival (DFS)]. We estimated outcomes using random effects meta-analysis to account for studies with high heterogeneity (*I*^2^ > 50 or *p* < 0.05). Otherwise, we used a fixed-effect model. We calculated odds ratios (ORs) for binary outcomes and standard mean differences (SMDs) for continuous outcomes. Treatment effects on OS and DFS were described by hazard ratio (HR).

**Results:**

This systematic review and meta-analysis of 15 studies on MIS vs. OT for NSCLC with N2 disease included 8,374 patients. Compared to OT, patients that underwent MIS had less estimated blood loss (EBL) (SMD = – 64.82, *p* < 0.01), shorter length of stay (LOS) (SMD = −0.15, *p* < 0.01), higher R0 resection rate (OR = 1.22, *p* = 0.049), lower 30-day mortality (OR = 0.67, *p* = 0.03), and longer overall survival (OS) (HR = 0.61, *P* < 0.01). The results showed no statistically significant differences in surgical time (ST), total lymph nodes (TLN), complications, and disease-free survival (DFS) between the two groups.

**Conclusion:**

Current data suggest that minimally invasive surgery may provide satisfying outcomes, a higher R0 resection rate, and better short-term and long-term survival than open thoracotomy.

**Systematic review registration:**

https://www.crd.york.ac.uk/PROSPERO/, identifier: CRD42022355712.

## 1. Introduction

Lung cancer is one of the most prevalent cancers worldwide and is mainly composed of non-small cell lung cancer (NSCLC), with nearly 1.8 million deaths globally in 2020 (1). Curative surgical resection is recommended as a frontline therapy for patients with early-stage NSCLC. Despite various treatments, including molecular targeting therapy, immunotherapy has gradually been used in clinical practice, and surgery is still recommended for NSCLC, particularly for patients in the early stage NSCLC (2–5). Mediastinal lymph node metastasis was closely related to a poor prognostic indicator of NSCLC. Patients with N2 NSCLC require precise evaluation of the mediastinum to provide optimal therapy, including chemotherapy, chemoradiotherapy (CRT), immunotherapy, and surgery (6–8). Recent studies suggested that MIS techniques provide safety and effectiveness compared to OT for patients with N2 NSCLC and may offer short-term and long-term advantages (9). In the past five decades, open thoracotomy (OT) has always been regarded as the standard surgical procedure for NSCLC. When Walker reported the first video-assisted thoracoscopic surgery (VATS) pneumonectomy (left) in 1994, VATS became more popular (10, 11). Furthermore, since Melfi reported the first robotic-assisted thoracic surgery (RATS) lobectomy in 2002 (12), the use of RATS has been widely applied in the aspects of thoracic surgery (13–15). The current study showed that MIS for N2 NSCLC might be associated with shorter and less ST compared to OT (16–18). The literature reported that MIS might not be inferior to OT in treating N2 NSCLC patients with neoadjuvant therapy (9, 19–21). Furthermore, most studies have shown comparable survival data and oncologic outcomes between the two surgical procedures (17–29). However, it is still unclear whether MIS is non-inferior to the OT approach in terms of safety and efficacy. After neoadjuvant therapy for N2 NSCLC, the tumor and mediastinal nodal stations may appear inflamed and have dense adhesions, which increase the risk of surgery (30, 31). Recently, a meta-analysis only analyzed the approach VATS and OT, which ignored the RATS approach (32). Therefore, we conducted a systematic review and meta-analysis to compare the effectiveness and safety of MIS and OT for N2 NSCLC.

## 2. Materials and methods

We report this systematic review and meta-analysis results according to the PRISMA 2020 statement (33) and registered with PROSPERO (CRD42022355712). We evaluated retrospective studies and randomized controlled trials with the Newcastle–Ottawa Scale (NOS) and Cochrane Risk Of Bias (ROB), respectively.

### 2.1. Search strategy

To identify relevant studies, we searched a systematic literature online database without time restriction, including PUBMED, EMBASE, SCOPUS, and WEB OF SCIENCE. We used the search terms combination [“robot” OR “robotic” AND “NSCLC” AND “N2”] with no restriction in language. We excluded conference abstracts, conference papers, and conference review publication types. The last search was run in August 2022. We manually searched the reference lists of retrieved articles to broaden the search.

### 2.2. Eligibility criteria

The study design we included was divided into three parts as follows: a retrospective study, a prospective study, and randomized controlled trials (RCTs). The study inclusion criteria were listed as follows: (1) patients with clinical or pathology diagnosed NSCLC with N2 disease; (2) surgical procedures that included at least two of the following simultaneously: RATS or VATS and OT; (3) at least one outcome evaluated, including conversion, surgical time (ST), estimated blood loss (EBL), R0 resection, length of stay (LOS), complications, total lymph nodes (TLN), disease-free survival (DFS), 30-day mortality, and overall survival (OS); and (4) no restriction in the language. The following exclusion criteria were considered for our study: (1) non-extractable data; (2) no relevant results; (3) single-arm studies; (4) not including RATS or VATS groups and OT groups; and (5) editorials, conference abstracts, and letters.

### 2.3. Data extraction

Two investigators (L.S.L. and S.P.L.) independently extracted data from eligible articles, and disagreements were resolved by discussion until reaching a consensus. The outcomes of interest were perioperative and long-term survival outcomes of the two surgical approaches (MIS vs. OT). The following information was extracted from each study: first author names, publication date, study center, mean age, sample size, surgical procedure, conversion, EBL, ST, TLN, LOS, neoadjuvant therapy, complications, 30-day mortality, disease-free survival (DFS), and overall survival (OS).

### 2.4. Data synthesis and analysis

Statistical analysis was performed using R software version 4.0.1. The “meta” package was used to evaluate all effect values. The standardized mean differences (SMDs), odds ratios (ORs), and hazard ratios (HRs) were used to assess continuous variables, dichotomous variables, and survival outcomes, respectively. For continuous data not presenting the means and standard deviations (SDs), we used McGrath et al.'s (34) method to estimate it. For studies without HR, we extract quality data from KM curves using the GetData Graph digitizer software and calculate HR using the methods of Guyot et al. (35). *I*^2^ and *p*-values were used to assess heterogeneity. For high heterogeneity (*I*^2^ ≥ 50% or *P* < 0.1), we used a random model to calculate this meta-analysis result and sensitive analysis. All results were conducted to analyze the RATS and VATS subgroups. All reported *p*-values were two-sided, and statistical significance was defined as *p* < 0.05.

## 3. Results

### 3.1. Systematic review and characteristics

**Figure 1:**
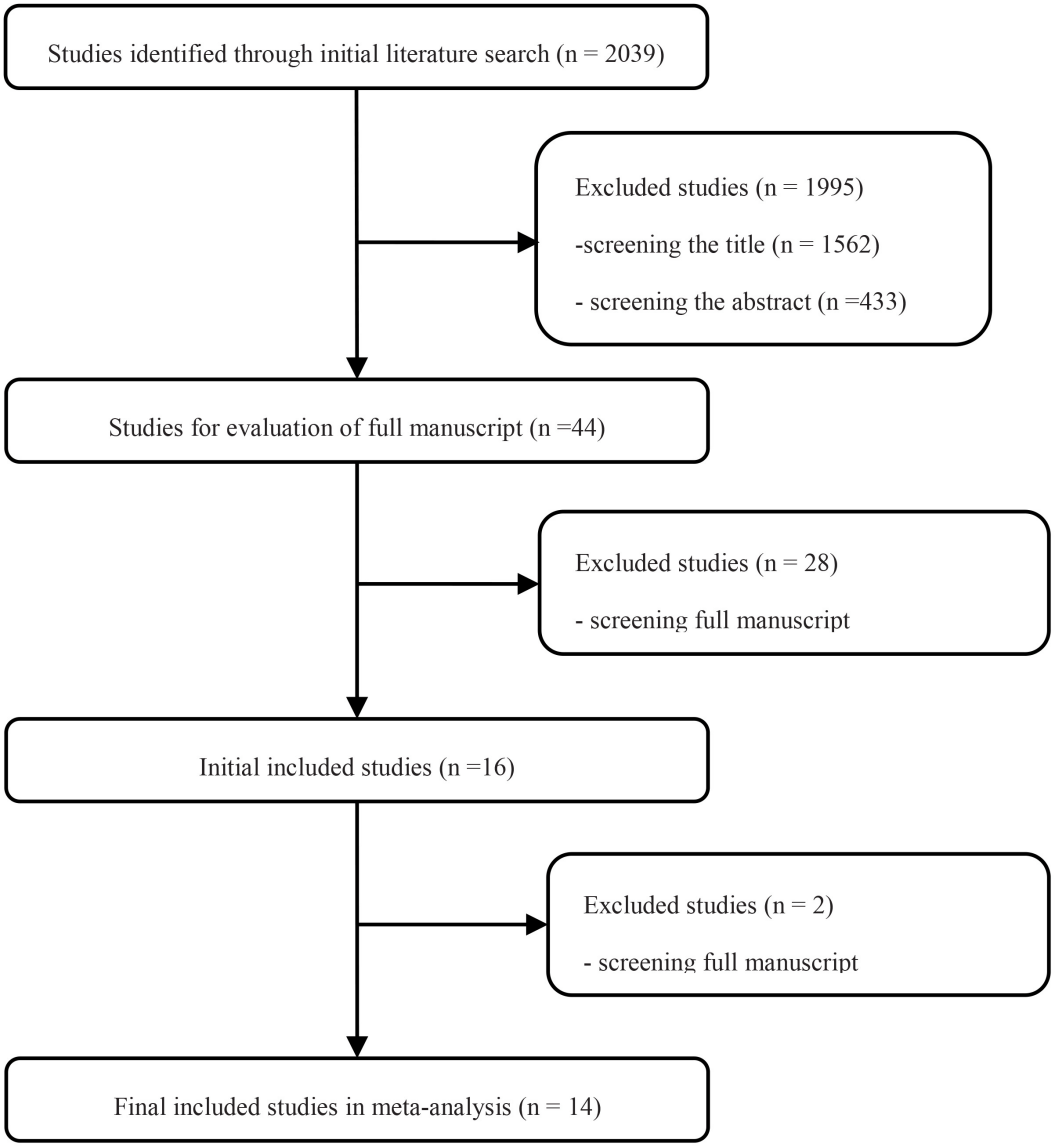
Flow diagram of study selection.

Searching the predefined search terms, we found 2,039 relevant records from five databases, and 43 studies were screened by hand. After the manual screening and eligibility assessment, 15 studies were included for further data analysis, including 8,347 patients. Finally, eight retrospective studies, five propensity-matched score studies, and two RCTs were included in the review (Figure 1). Detailed clinical information of each study is presented in Table 1.

**Table 1:** Baseline data for studies included in the meta-analysis.

**Author**	**Year**	**Study**	**Center**	**Procedure**	**Sample**	**Mean, age**	**Neoadjuvant therapy**	**Quality scores**
Jeon	2021	PSM	Single center	VATS	143	62	100%	8
Casiraghi	2021	PSM	Single center	RATS	32	NA	100%	8
Liu	2020	PSM	Single center	VATS	592	57	4%	8
Huang	2021	RCT	Multicenter	RATS	148	60.9	0%	5
Herb	2020	Retrospective	Multicenter	MIS	5,741	65	47%	8
Zhao	2020	PSM	Multicenter	VATS	338	58.78	8.20%	8
Yun	2020	IPTM	Single center	VATS	268	63.8	0%	8
Huang	2019	RCT	Multicenter	RATS	113	61.9	0%	5
Yamashita	2019	Retrospective	Single center	VATS	79	67.16	NA	7
Jeon	2017	Retrospective	Single center	VATS	35	62.7	100%	7
Zhong	2013	Retrospective	Single center	VATS	157	59.6	0%	7
Li	2012	Retrospective	Single center	VATS	76	64	0%	7
Zhou	2011	Retrospective	Single center	VATS	263	58	0%	7
Watanabe	2007	Retrospective	Single center	VATS	69	68	0%	7
Wang	2013	Retrospective	Single center	VATS	320	57.6	0%	8

### 3.2. Intraoperative outcomes

#### 3.2.1. Conversion

**Figure 2:**
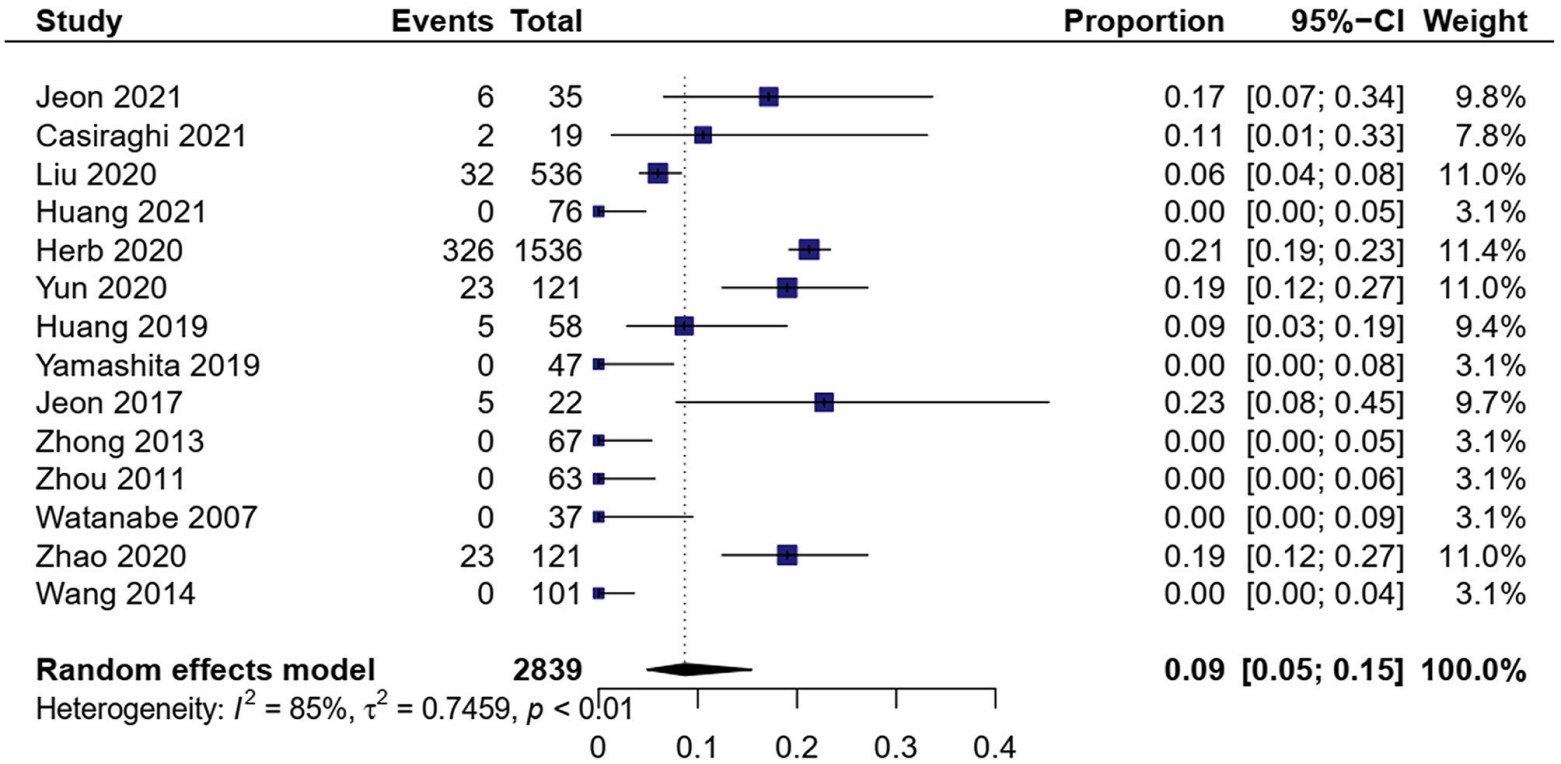
Forest plots of open conversion in minimally invasive surgery (MIS).

A total of 14 studies showed MIS conversion to OT in 2,839 patients (9, 16, 17, 19–29). The meta-analysis result of heterogeneity was high (*I*^2^ = 84.7%, *p* < 0.01). The total proportion of MIS was 8.7% (0.05, 0.15) (Figure 2). Subgroup analysis showed that the proportion of RATS conversion to open surgery was 9.2% (0.07, 0.12) and VATS conversion to open surgery was 8.2% (0.04, 0.17). RATS had a little higher conversion than VATS [OR = 1.97, 95% CI (1.44, 2.69), *P* < 0.01].

#### 3.2.2. EBL

**Figure 3:**
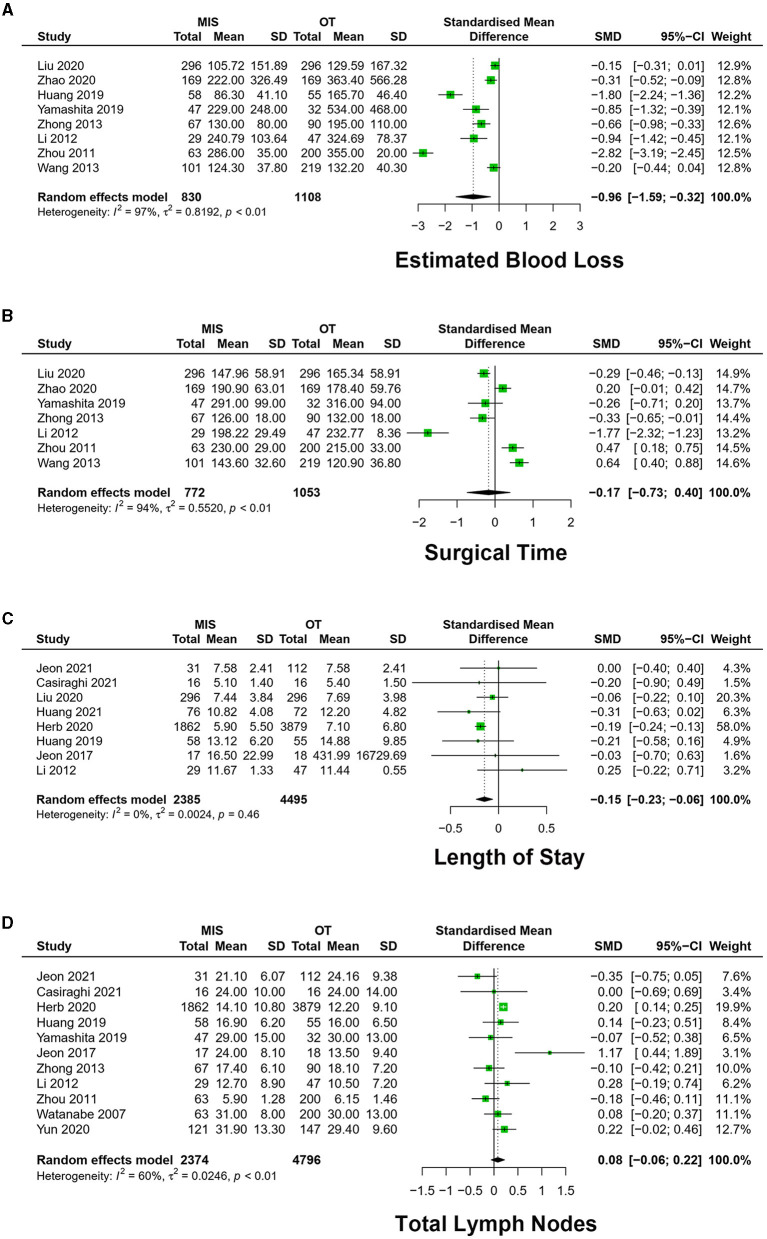
Forest plots of **(A)** estimated blood loss, **(B)** surgical time, **(C)** length of stay, and **(D)** total lymph nodes. MIS, minimally invasive surgery; VATS, video-assisted thoracoscopic surgery; CI, confidence interval.

Eight studies exhibited estimated blood loss in the included 1,938 patients (16–18, 22, 24, 26, 27, 29). The meta-analysis result of heterogeneity was low (*I*^2^ = 96.7%, *p* < 0.01). MIS had less EBL than OT [SMD = −0.96, 95% CI (−1.59, −0.32), *p* = 0.003] (Figure 3A).

#### 3.2.3. ST

A total of 10 studies exhibited surgery time in 2,118 patients (16–18, 20, 22, 23, 26, 27, 29). The meta-analysis result of heterogeneity is high (*I*^2^ = 91.9%, *p* < 0.01). There was no significant difference in ST [SMD = −0.01, 95% CI (– 0.45, 0.47), *P* = 0.93] (Figure 3B).

Seven studies exhibited VATS vs. OT, with a high degree of heterogeneity (*I*^2^ = 93.9%, *P* < 0.01), which has no significant difference in ST [SMD = −0.17, 95% CI (– 0.73, 0.40), *P* = 0.56].

**Figure 4:**
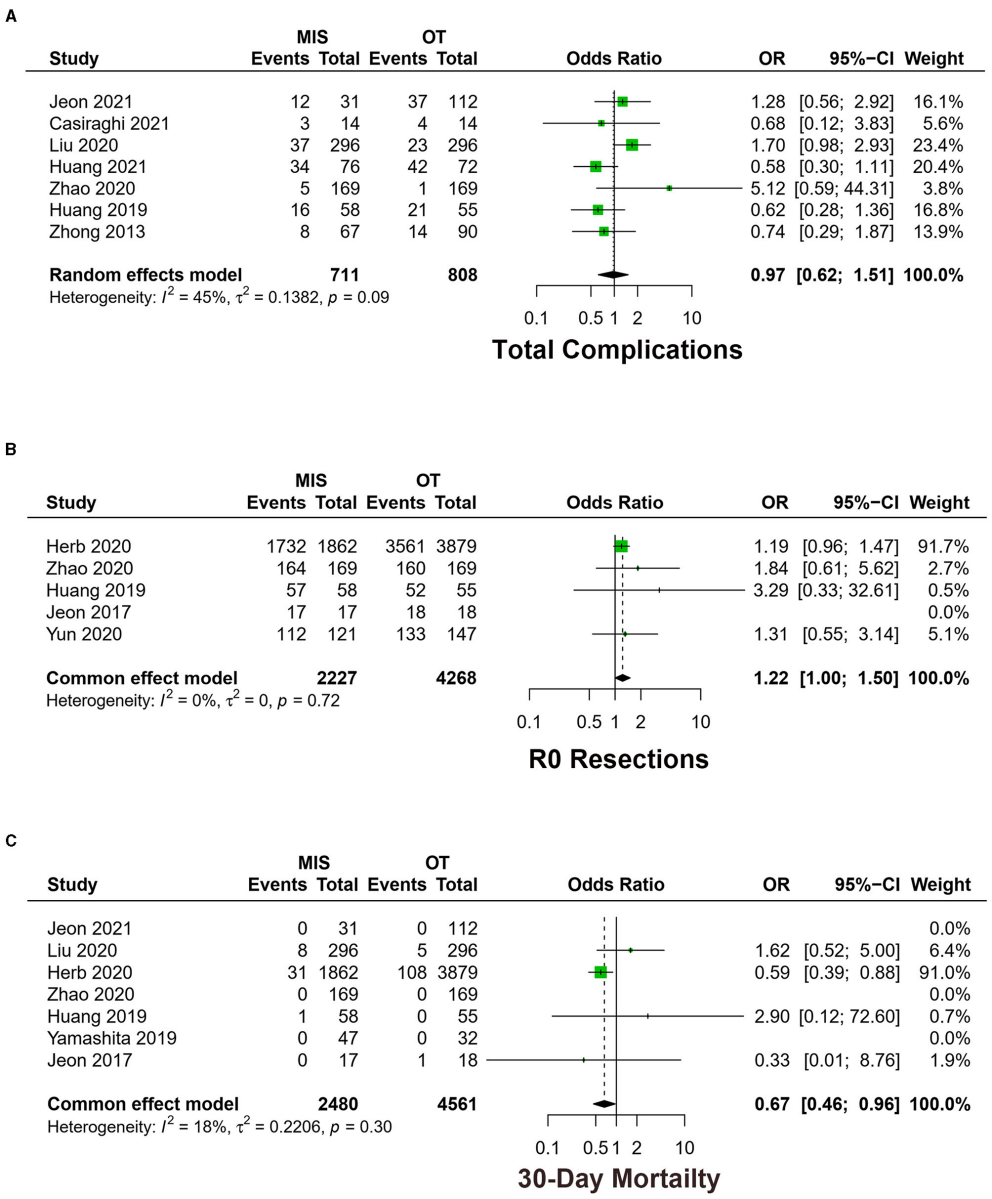
Forest plots of **(A)** total complications, **(B)** R0 resections, and **(C)** 30-day mortality between MIS and OT. MIS, minimally invasive surgery; VATS, video-assisted thoracoscopic surgery; CI, confidence interval.

Three studies exhibited RATS vs. OT, with a high degree of heterogeneity between studies (*I*^2^ = 78.3%, *p* = 0.01), and ST was not statistically significantly different in the two groups [SMD = 0.44, 95% CI (−0.30, 1.17), *p* = 0.24] (Figure 4).

#### 3.2.4. TLN

A total of 11 studies exhibited the number of total lymph nodes in dissection among 7,170 patients (9, 16–21, 26–28). The meta-analysis result of the heterogeneity was high between studies (*I*^2^ = 60.3%, *p* < 0.01). Total lymph nodes (TLN) have no significant difference between the MIS and OT groups [SMD = 0.08, 95% CI (−0.06, 0.22), *P* = 0.25] (Figure 3D).

The heterogeneity of nine studies was high (*I*^2^ = 66.1%, *P* < 0.01) among studies between VATS and OT, with no significant difference in TLN [SMD = 0.07, 95% CI (−0.09, 0.24), *P* = 0.38].

Three studies exhibited RATS vs. OT, which showed a low degree of heterogeneity between studies (*I*^2^ = 0.0%, *p* = 0.69). RATS had more TLN [SMD = 0.23, 95% CI (0.14, 0.33), *P* < 0.01].

### 3.3. Postoperative outcomes

#### 3.3.1. LOS

A total of eight studies exhibited the length of hospital stay among 6,880 patients (9, 16, 18–23). The meta-analysis result of heterogeneity was high among studies (*I*^2^ = 0.0%, *p* = 0.46). MIS had less LOS [SMD = −0.15, 95% CI (−0.23, −0.06), *p* < 0.01] (Figure 3C).

Five studies exhibited low heterogeneity (*I*^2^ = 7.9%, *p* = 0.36) between VATS and OT. VATS had shorter LOS [SMD = −0.10, 95% CI (−0.20, −0.001), *p* = 0.046].

Four studies reported RATS vs. OT, and the heterogeneity is high among studies (*I*^2^ = 0.0%, *p* = 0.96). RATS had shorter LOS [SMD = −0.30, 95% CI (−0.35, −0.25), *p* < 0.01].

#### 3.3.2. Complications

Seven studies exhibited total complications among 1,519 patients (16, 18–20, 22–24). The meta-analysis result of heterogeneity is low (*I*^2^ = 44.8%, *p* = 0.09), with no difference between MIS and OT [OR = 0.97, 95% CI (0.60, 1.51), *p* = 0.89] (Figure 4A).

Four studies exhibited total complications of VATS vs. OT, which is a low heterogeneity among studies (*I*^2^ = 19.5%, *p* = 0.29). No statistically significant difference was identified between the two groups [OR = 1.40, 95% CI (0.97, 2.13), *p* = 0.07].

Three studies exhibited total complications of RATS vs. OT, and the meta-analysis result of heterogeneity was low (*I*^2^ = 0.0%, *p* = 0.98). RATS had fewer complications [OR = 0.60, 95% CI (0.37, 0.97), *p* = 0.04].

#### 3.3.3. R0 resections

Five studies exhibited the rate of R0 resections, including 6,495 patients (9, 16, 21, 24, 25). The meta-analysis result of the heterogeneity is low among studies (*I*^2^ = 0.0%, *p* = 0.72). MIS had higher R0 resections [OR = 1.22, 95% CI (1.00, 1.50), *p* < 0.05] (Figure 4B).

Three studies exhibited R0 resections of VATS vs. OT, and the meta-analysis result of heterogeneity between the studies was low (*I*^2^ = 0.0%, *p* = 0.40). There was no statistical difference in R0 resections between VATS and OT [OR = 1.15, 95% CI (0.92, 1.44), *p* = 0.22].

Two studies exhibited R0 resections of RATS vs. OT, and the heterogeneity in each study was low (*I*^2^ = 0.0%, *p* = 0.48). No statistically significant difference was identified in R0 resections in the two groups [OR = 1.47, 95% CI (0.98, 2.19), *p* = 0.06].

### 3.4. Survival outcomes

#### 3.4.1. 30-day mortality

Seven studies exhibited 30-day mortality (9, 16, 19, 21, 22, 24, 26). The meta-analysis result of heterogeneity is low (*I*^2^ = 18.4%, *p* = 0.30). MIS had lower 30-day mortality [OR = 0.67, 95% CI (0.46, 0.96), *p* = 0.03] (Figure 4C).

Six studies exhibited 30-day mortality of VATS vs. OT, which showed low heterogeneity among studies (*I*^2^ = 8.5%, *p* = 0.34). There was no statistical difference in 30-day mortality in VATS and OT groups [OR = 0.75, 95% CI (0.51, 1.11), *p* = 0.15].

Two studies exhibited 30-day mortality of RATS vs. OT, and the meta-analysis result of heterogeneity was low (*I*^2^ = 41.2%, *p* = 0.19). The RATS group had lower 30-day mortality [OR = 0.36, 95% CI (0.15, 0.90), *p* = 0.03].

#### 3.4.2. OS

**Figure 5:**
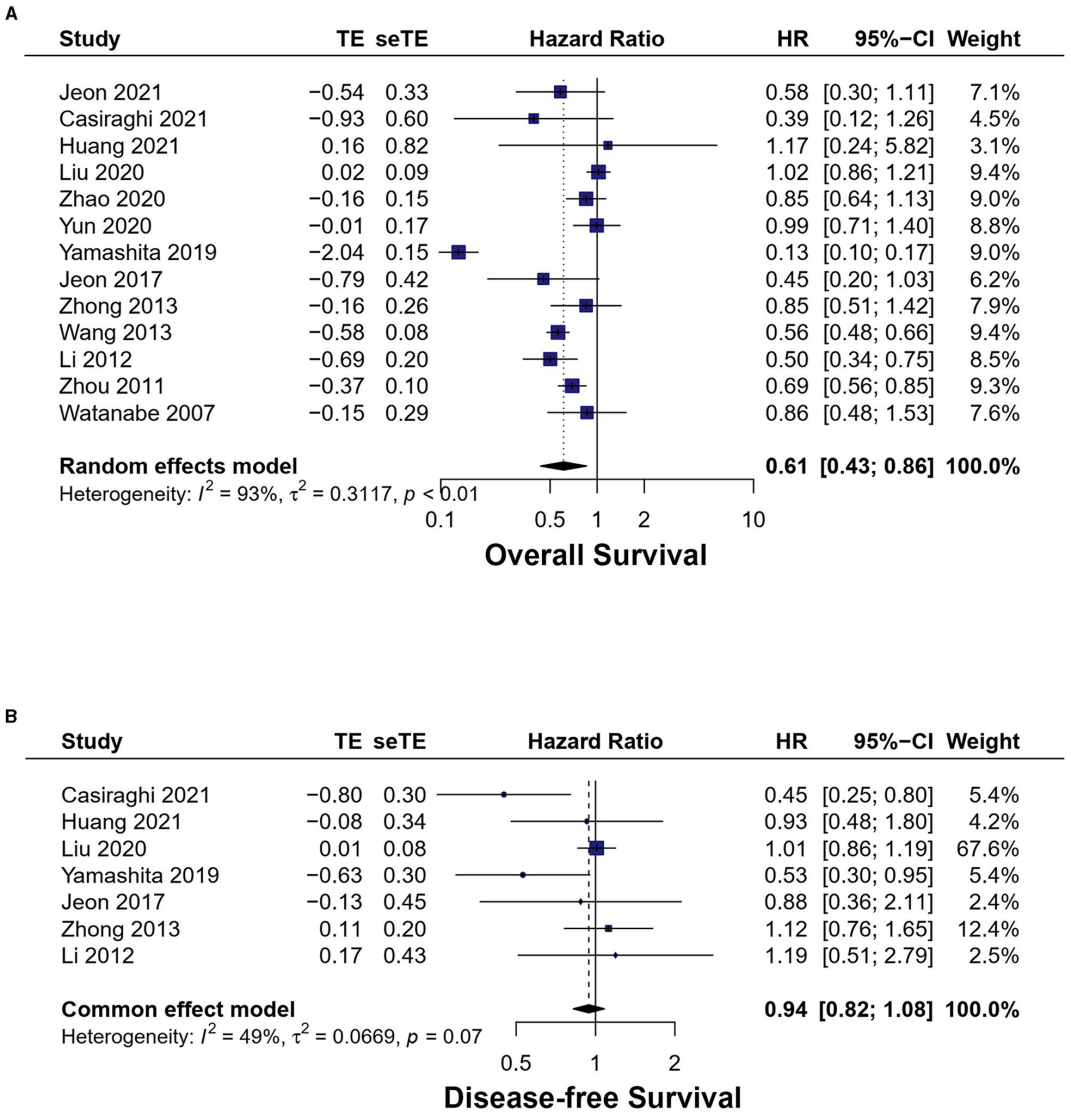
Forest plots of **(A)** overall survival, **(B)** disease-free survival. MIS, minimally invasive surgery; VATS, video-assisted thoracoscopic surgery; CI, confidence interval.

A total of 13 studies exhibited overall survival (17–29). The meta-analysis result of the heterogeneity test is high (*I*^2^ = 92.8%, *p* < 0.01). MIS had longer OS [HR = 0.61, 95% CI (0.43, 0.86), *p* < 0.01] (Figure 5A).

In total, 11 studies exhibited overall survival of VATS vs. OT and high heterogeneity between studies (*I*^2^ = 94%, *p* < 0.01). VATS had longer OS [HR = 0.61, 95% CI (0.42, 0.88), *p* < 0.01].

Two studies exhibited overall survival of RATS vs. OT, which showed a low heterogeneity (*I*^2^ = 14.30%, *p* = 0.28). No statistically significant difference was identified in OS between the two groups [HR = 0.57, 95% CI (0.22, 1.48), *p* = 0.25].

Nine studies reported overall survival without neoadjuvant therapy and the MIS had longer OS [HR = 0.77, 95% CI (0.63, 0.93), *p* < 0.01]. Three studies reported overall survival with neoadjuvant therapy and the MIS had longer OS [HR = 0.50, 95% CI (0.31, 0.80), *p* < 0.01].

#### 3.4.3. Disease-free survival

Seven studies reported disease-free survival (17, 18, 20–23, 26). The meta-analysis result of the heterogeneity test is low (*I*^2^ = 33.7%, *p* = 0.17). No statistically significant difference was found between the two groups [HR = 0.94, 95% CI (0.82, 1.08), *p* = 0.38] (Figure 5B).

Five studies reported disease-free survival of VATS vs. OT, which is a low heterogeneity between studies (*I*^2^ = 17.8%, *p* = 0.30). No statistically significant difference was found between VATS and OT [HR = 0.98, 95% CI (0.85, 1.13), *p* = 0.80].

Two studies reported disease-free survival of RATS vs. OT, and the heterogeneity was low (*I*^2^ = 20.4%, *p* = 0.26). There was no statistical difference between RATS and OT [HR = 0.68, 95% CI (0.46, 1.01), *p* = 0.06].

### 3.5. Publication bias and sensitivity analysis

**Figure 6:**
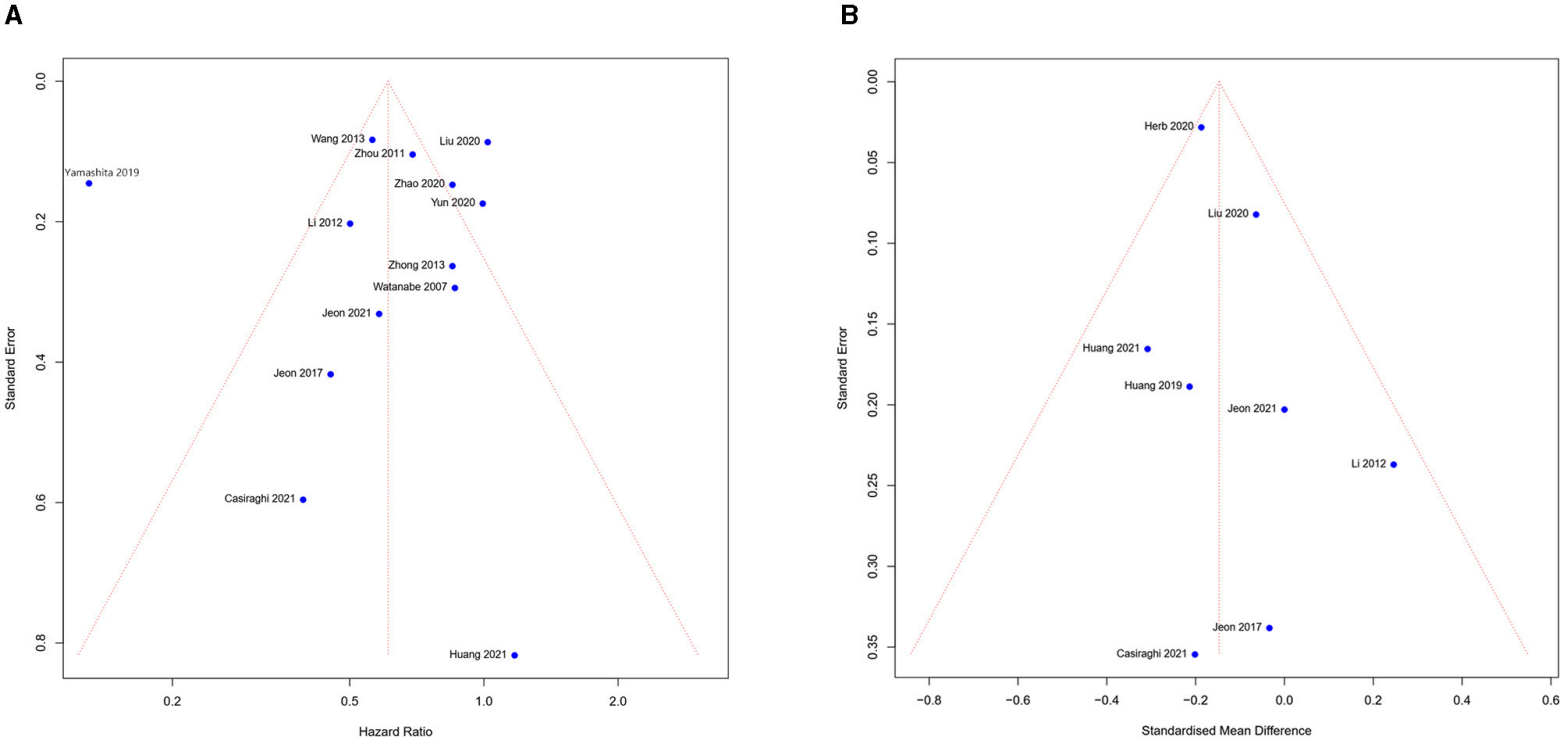
Funnel plots of publication bias test: **(A)** overall survival and **(B)** length of stay.

The funnel chart was used to assess publication bias. According to the overall survival and length of stay (Figures 6A, B), we noticed the study was symmetrically distributed in the funnel chart, which suggests that there was no significant publication bias in this meta-analysis. Although the quality of all studies we included was high, some heterogeneity of studies from different countries was inevitable. We used sensitivity analysis to find the possible source for the meta-analysis result with high heterogeneity between studies. The meta-analysis result showed high heterogeneity (conversion, EBL, ST, TLN, and OS), thus we excluded each study and analyzed the result individually. If the results do not change significantly, we consider the results stable. Finally, all results showed a steady source of heterogeneity.

## 4. Discussion

For the past five decades, open thoracotomy (OT) has been regarded as the preferred surgical procedure for patients with NSCLC. However, when Walker (10) reported the first VATS for pneumonectomy, VATS gradually became the common standard approach for NSCLC. Recently, there has been a rapid emergence of minimally invasive and highly efficacious Robotic Assisted Technologies, promptly capturing the attention of the majority of surgeons. A considerable proportion of N2 NSCLC patients often opt for an initial course of neoadjuvant therapy, followed by subsequent surgical intervention, as a means to attain enhanced oncological outcomes and improved long-term survival. Although recently, a meta-analysis (32) reported VATS vs. OT for the management of N2 NSCLC, they did not include the surgery approach of RATS and the patients of neoadjuvant therapy, which is not consistent with clinical practice. For locally advanced N2 NSCLC, the National Comprehensive Cancer Network (NCCN) guidelines suggested neoadjuvant therapy and surgery. After neoadjuvant therapy, the tumor and tissue appear inflamed and have dense adhesions (31), which may increase the risk of surgery. Therefore, we performed this meta-analysis to compare the safety and effectiveness of MIS and OT in N2 NSCLC. Our meta-analysis pooled the currently available studies and compared the perioperative and long-term survival outcomes of MIS and OT for NSCLC with N2 disease.

Our meta-analysis demonstrated that the MIS group had less EBL (SMD = −0.96, *p* = 0.003), shorter LOS (SMD = −0.15, *P* < 0.01), and low 30-day mortality (OR = 0.67, *p* = 0.03) but no difference in ST (SMD = −0.01, *p* = 0.93) compared to the OT group. The high heterogeneity between studies mainly came from differences in different countries and centers, subjective bias in the statistical blood, and differences in surgeon proficiency. The same result was observed in LOS and ST in the RATS and VATS subgroup analyses. Minimally invasive surgery has the advantages of inducing less trauma, a wider field of vision, less bleeding, and fewer hospital stays from previous studies (36–38). Due to defects, a lack of direct touch, limitations of surgical instruments, and other factors, MIS is longer than OT with surgical time. However, with the accumulating experience of surgeons, the RATS and VATS time can be further shortened (39). Herb et al. (9) retrospectively analyzed 5,741 N2 NSCLC patients between the MIS group and OT group, which demonstrated that the MIS group had a higher R0 resection rate but no significance. In a retrospective study of 770 cN0-pN2 NSCLC patients (450 VATS, 320 open), Watanabe et al. (28) found no differences in total lymph node count, lymph node station count, mediastinal lymph nodes, or mediastinal station count between VATS and OT. Our study found that the MIS group had a marginally significant difference than the OT group in the R0 resection rate [OR = 1.22, 95% CI (1.00, 1.50), *P* = 0.049]. However, there was no significant R0 resection rate in the RATS and VATS subgroups. The MIS group had no difference in TLN (SMD = 0.08, *P* = 0.25). However, subgroup analysis showed that the RATS group had more TLN [SMD = 0.23, 95% CI (0.14, 0.33), *P* < 0.01]. The previous studies of some small samples showed no difference between the MIS and OT groups (16, 19, 20). Nevertheless, Herb et al. (9) studied a large sample and reported that the MIS group could dissect more TLN, and a subgroup analysis presented the same results. RATS, with a wider field of view and more flexible operation in a narrow space than VATS, can explain why MIS may dissect more lymph nodes and has higher R0 resection rate (40, 41). The MIS group had longer OS (HR = 0.61, *p* < 0.01) but no difference in DFS. Subgroup analysis divided by surgery approach and neoadjuvant therapy showed the same result. Wang et al. (29) reported that the VATS group had a better 5-year OS than the OT group for N2 NSCLC. Yamashita et al. (26) found that the VATS approach following neoadjuvant treatment had better OS and no significance in DFS for the treatment of stage IIIA N2 NSCLC. Several studies have demonstrated that VATS reduced postoperative inflammatory response and impaired immunity more than OT, contributing to less complication and better long-term outcomes (42–44). Moreover, the MIS group had a little high R0 resection in our meta-analysis. Above all, these factors could potentially account for the MIS group exhibiting a longer OS. Due to practical constraints, this meta-analysis has several limitations. First, most studies we included were non-randomized and controlled trials. Only four studies had the RATS group, and most studies reported the VATS group. Second, the majority of these studies were conducted in China. Therefore, further research is warranted to ascertain the applicability of these findings to the context of Western countries. Third, we included N2 diseases diagnosed by radiography and pathology, which may result in inconsistent postoperative pathological staging between the two groups. This mainly affects the postoperative survival time. Therefore, we performed the pathological staging subgroup to make it up. However, when the preoperative pathology was N2 NSCLC and the postoperative pathology changed, we failed to perform further subgroup analysis according to the postoperative pathological stage. Therefore, more high-quality randomized trials need to be conducted in the future.

## 5. Conclusion

Minimally invasive surgery had advantages compared to OT in the management of N2 NSCLC in terms of shorter LOS, less EBL, low 30-day mortality, high R0 resection, and longer OS, while ST and complications were similar. However, the limitations and scanty evidence of the included studies still require more randomized controlled trials with high quality and larger sample sizes to be demonstrated.

## Data availability statement

The raw data supporting the conclusions of this article will be made available by the authors, without undue reservation.

## Author contributions

SLiu and GQ conceived and designed the study. SLiu, SLi, and YT contributed to the analysis. SLiu and RC wrote the manuscript. All authors have read and approved the final manuscript. All data can be made available upon request to the author.
